# The glycoprotein of vesicular stomatitis virus promotes release of virus-like particles from tetherin-positive cells

**DOI:** 10.1371/journal.pone.0189073

**Published:** 2017-12-07

**Authors:** Constantin Brinkmann, Markus Hoffmann, Anastasia Lübke, Inga Nehlmeier, Annika Krämer-Kühl, Michael Winkler, Stefan Pöhlmann

**Affiliations:** Infection Biology Unit, German Primate Center, Kellnerweg 4, Göttingen, Germany; Cincinnati Children's Hospital Medical Center, UNITED STATES

## Abstract

Vesicular stomatitis virus (VSV) release from infected cells is inhibited by the interferon (IFN)-inducible antiviral host cell factor tetherin (BST-2, CD317). However, several viruses encode tetherin antagonists and it is at present unknown whether residual VSV spread in tetherin-positive cells is also promoted by a virus-encoded tetherin antagonist. Here, we show that the viral glycoprotein (VSV-G) antagonizes tetherin in transfected cells, although with reduced efficiency as compared to the HIV-1 Vpu protein. Tetherin antagonism did not involve alteration of tetherin expression and was partially dependent on a GXXXG motif in the transmembrane domain of VSV-G. However, mutation of the GXXXG motif did not modulate tetherin sensitivity of infectious VSV. These results identify VSV-G as a tetherin antagonist in transfected cells but fail to provide evidence for a contribution of tetherin antagonism to viral spread.

## Introduction

Vesicular stomatitis virus (VSV) is a negative-stranded RNA virus within the *Rhabdoviridae* family, and VSV New Jersey and Indiana are major VSV serotypes. VSV is transmitted from insects to ungulates (mainly cattle, horses and pigs), in which it can cause mucosal lesions [1–3]. In addition, the virus can be transmitted to humans and such infections usually induce influenza-like symptoms [3]. VSV replicates fast, is highly immunogenic and is frequently used to model infection by negative-stranded RNA viruses. Moreover, VSV is used as a tool for diverse scientific endeavors [4]. For instance, VSV has oncolytic properties [5] and is developed for cancer therapy [6]. Moreover, VSV variants in which the open reading frame for the viral glycoprotein (VSV-G) has been replaced by that of the Ebola virus (EBOV) glycoprotein (GP) are currently tested as vaccines against EBOV infection [7–9].

The interferon (IFN) system is an integral component of innate immunity and constitutes the first line of defense against viral infection. Sensors of the IFN system, including toll-like receptors and retinoic acid inducible gene I-like receptors, can detect pathogen-associated molecular patterns (PAMPs), which triggers signals that commandeer the cells to express IFN [10,11]. Binding of IFN to uninfected cells in turn triggers further signaling events that induce the expression of IFN-stimulated genes (ISG), many of which exert antiviral activity [12,13]. VSV spread can be blocked by IFN in cell culture, although the viral matrix protein VSV-M interferes with IFN signaling [14–16]. The ISG-encoded proteins that are responsible for IFN-induced blockade of VSV infection are not fully known, although IFITM3 and tetherin were shown to block VSV infection in transfected cells [17,18].

The IFN-induced antiviral host cell protein tetherin (CD317, BST-2) blocks release of diverse enveloped viruses from infected cells [19,20]. The particular membrane topology of tetherin is key to its antiviral activity: Tetherin harbors an N-terminal transmembrane domain and a C-terminal GPI-anchor which allows the protein to simultaneously insert into viral and cellular membranes, thereby forming a physical tether between virus and host cell [21]. Several viruses encode tetherin antagonists which allow viral spread in tetherin-positive cells [22]. The prototypic tetherin antagonist, the HIV-1 protein Vpu, and most other viral tetherin antagonists block tetherin by reducing its expression at the plasma membrane [23–25], which is used by these viruses as platform for budding of progeny particles. In contrast, the EBOV-GP, another tetherin antagonist, interferes with tetherin’s antiviral activity without modulating tetherin expression or cellular localization [26–29] and the mechanism underlying tetherin antagonism by EBOV-GP is largely unclear. Two studies reported that VSV is inhibited by tetherin. Weidner and colleagues showed that directed expression of tetherin resulted in a profound decrease in VSV release from infected cells [18]. Liberatore and coworkers dissected cell-cell spread of VSV from viral dissemination to distal cells via free particles and found that only the latter process was markedly inhibited by tetherin [17]. However, it is at present unknown whether VSV encodes a tetherin antagonist, which is responsible for residual viral spread in tetherin-positive cells.

Here, we show that VSV-G counteracts tetherin in transfected cells. However, no evidence for a contribution of tetherin-antagonism to spread of authentic VSV in tetherin-positive cells was obtained.

## Material and methods

### Cell lines and transfection

Human embryonal kidney-293T, Vero (African green monkey, kidney) and HeLa (human, cervix carcinoma) cells were maintained in Dulbecco’s Modified Eagle Medium (DMEM) supplemented with 10% fetal bovine serum (FBS, Biochrome, Berlin) and penicillin/streptomycin (PAN-Biotech, Aidenach; final concentration penicillin 100 units/ml, streptomycin 0.1 µg/ml). BHK-21 cells (baby hamster kidney) were cultivated in DMEM supplemented with 5% FBS (Biochrome) and penicillin/streptomycin. Cells were cultured at 37°C in humidified atmosphere containing 5% CO_2_. For seeding and subcultivation, cells were washed with phosphate-buffered saline (PBS) and detached by incubation in a trypsin/EDTA solution (PAN-Biotech, Aidenach; HeLa, Vero and BHK-21 cells) or by directly resuspending them in DMEM (293T). Cell numbers were determined under a light microscope using a Neubauer chamber. Transfection of Vero, BHK-21 and HeLa cells was performed using Lipofectamine2000 (ThermoFisher Scientific, Dreieich) according to manufacturer’s protocol, while 293T cells were transfected using the calcium phosphate method. Selection and maintenance of Vero cells stably transfected with the retroviral pQCXIP vector was achieved using puromycin (Cayman Chemical, Ann Arbor). 293T cells were obtained from DSMZ (ACC-635, Leibniz Institute DSMZ–German Collection of Microorganisms and Cell Cultures). Vero and BHK-21 cells were obtained from collaborators. HeLa cells were obtained through the NIH AIDS Reagent Program, Division of AIDS, NIAID, NIH and their identity was confirmed by STR DNA typing employing a published protocol [30,31].

### Plasmids

Plasmids encoding Vpu, EBOV-GP and HIV-1 p55-Gag were previously described [27,32]. Expression plasmids for the coding sequences of VSV nucleoprotein (VSV-N), phosphoprotein (VSV-P), matrix protein (VSV-M), glycoprotein (VSV-G) and RNA-dependent RNA-polymerase (VSV-L) were generated by amplifying the respective open reading frame (ORF) from a plasmid-encoded VSV anti-genome (Indiana strain, kindly provided by G. Zimmer) and inserting them via standard cloning procedures into plasmids pCAGGS (VSV-N, VSV-P, VSV-M (mutant ncp, harboring four amino acid substitutions associated with reduced cytotoxicity [33]) and VSV-L) or pCG1 (VSV-G). This was achieved by overlap-extension PCR technique using primers that introduce the desired nucleotide exchanges. Generation of VSV-G mutants A133R and LXXXL was achieved by the same strategy using the expression plasmid for wt VSV-G as a template. The expression plasmid for human tetherin was generated by amplifying the ORF from a previously described plasmid [34] and inserting it into plasmid pCAGGS using KpnI and XhoI restriction sites. Similarly, porcine tetherin was PCR amplified and cloned via EcoRI and NheI restriction sites. For detection of human tetherin expression, the sequence for a c-Myc antigenic tag was added to the 5’ end of human tetherin using PCR. In order to generate transgenic cells stably expressing human tetherin, the genetic information for human tetherin was cloned into the retroviral vector pQCXIP-mcs, a modified form of pQCXIP (Clontech, Palo Alto), in which the multiple cloning site was modified to contain sites for NotI-BamHI-AgeI-HpaI-MluI-XhoI-NruI-EcoRI. To generate recombinant VSV that expresses eGFP (enhanced green fluorescent protein), we constructed a plasmid-encoded VSV anti-genome (GenBank: J02428.1) with an additional transcription unit for eGFP between VSV-G and VSV-L ORFs. First, unique MluI and NheI restriction sites were introduced upstream and downstream of the ORF for VSV-G, respectively. Next, a cassette consisting of the coding sequences of VSV-G and eGFP, separated by a minimal intergenic region [35] was cloned and inserted into the parental VSV genome making use of MluI and NheI restriction sites, thereby replacing the genetic information of VSV-G and thus generating VSV* (the asterisk stands for eGFP). For convenient exchange of VSV-G or eGFP ORFs by other transcription units, unique AscI and NotI restriction sites were added upstream and downstream of the VSV-G and eGFP ORFs, respectively. In order to generate VSV* mutant VSV-G (LXXXL), the respective ORF was amplified from the corresponding VSV-G (LXXXL) expression plasmids with primers adding 5’ MluI and 3’ AscI restriction sites and inserted into VSV*, thereby replacing the parental ORF coding for wt VSV-G. The integrity of all PCR-amplified sequences was confirmed by automated sequencing.

### Virus-like particle-based release assay

Release of virus-like particles (VLPs) and its inhibition by tetherin has been examined as described [27,32]. In brief, 293T cells were seeded in 6-well plates and cotransfected with plasmids encoding HIV-1 p55-Gag, tetherin and a potential tetherin antagonist or empty plasmid, using the calcium phosphate method. At 16 h post transfection, the transfection medium was replaced by fresh culture medium. For blockade of VSV-G-dependent tetherin antagonism, anti-VSV-G hybridoma supernatant (I1, concentrated mouse hybridoma supernatant from CRL-2700, ATCC) was added to the culture medium at a final dilution of 1:1,000. At 48 h post transfection the supernatants were collected and cells were lysed in 200 μl of 2x SDS-containing lysis buffer (30 mM Tris [pH 6.8], 10% glycerol, 2% SDS, 5% β-mercaptoethanol, 0.1% bromophenol blue, 1mM EDTA). The lysates were incubated at 95°C for 30 min. The supernatants were cleared by centrifugation and VLPs were pelleted from cleared supernatants by centrifugation through a 20% sucrose cushion. The concentrated VLPs were lysed in 50 μl 2 x SDS loading buffer and incubated at 95°C for 30 min. Subsequently, cell lysates and supernatants were analyzed for the presence of Gag employing Western blot. For immunoblotting, the proteins were separated via SDS-polyacrylamid (PAA) gel electrophoresis using a 12.5% PAA gel and transferred onto a nitrocellulose membrane (GE Healthcare Life Sciences, Solingen, 0.2 μm). The membranes were blocked in 5% milk powder in PBS supplemented with 0.1% Tween 20 and Gag-protein was detected using 1:100 diluted supernatants of hybridoma cells secreting a mouse anti-Gag antibody (183-H12-5C). Expression of VSV-G wt and mutants was detected using the aforementioned anti-VSV-G hybridoma supernatant at a dilution of 1:100 while VSV-M was detected using mouse monoclonal antibody 23H12 (Kerafast, Boston). Tetherin expression was detected employing anti c-Myc-hybridoma supernatants (C-Mycl-9E10 (ECACC 85102202)). Expression of VSV proteins, Vpu and EBOV-GP was detected using previously described rabbit sera [36–38]. Expression of ß-actin was detected using mouse anti-ß-actin antibody (Sigma-Aldrich, Munich) at a dilution of 1:1,000. Bound antibodies were detected using HRP-coupled anti-mouse and anti-rabbit secondary antibodies (Dianova, Hamburg) at a final concentration of 0.1 μg/ml. Binding of secondary antibodies was detected using a self-made ECL solution (0.1 M Tris-HCl pH 8.6, 250 μg/ml Luminol (Sigma-Aldrich, München), 1 mg/ml para-hydroxycomaric acid (Sigma-Aldrich, München); 0.3% H_2_O_2_) and signals were visualized using the ChemoCam imaging system and the ChemoStarProfessional software (Intas). For quantification of the signal intensity, the program ImageJ (FIJI distribution) [39] was used. Gag signals detected in the supernatants were normalized against the respective signals obtained in cell lysates.

### Rescue and quantification of recombinant VSV

For the rescue of VSV* harboring wt or mutant VSV-G, we employed a strategy developed by others [40] with slight modifications. First, BHK-21 cells were grown in 12-well dishes until they reached ~70% confluency. At this point, the cells were infected with recombinant modified vaccinia virus Ankara expressing T7 polymerase ([41], vMVA-T7, kindly provided by G. Sutter) at a multiplicity of infection (MOI) of 3. At 1 h post infection, the inoculum was removed and the cells were transfected with expression plasmids for VSV-N, -P and -L (see above) as well as the respective, plasmid-encoded VSV* anti-genome (the genome is preceded by a T7 promotor sequence and followed by a hepatitis delta virus ribozyme and a T7 terminator sequence for cytoplasmic production of negative-sensed viral genomes that are template for transcription and genome replication) using Lipofectamine2000 (ThermoFisher Scientific, Dreiech) as transfection reagent. The following DNA concentrations were used (per well): 0.4 μg VSV-N, 0.35 μg VSV-P, 0.25 μg VSV-L and 1.0 μg plasmid-encoded VSV* or VSV*(LXXXL) genome. Transfection was carried out in OptiMEM medium (ThermoFisher Scientific, Dreiech) for 6 h, before the transfection medium was replaced by standard culture medium. At 12 h post transfection, the culture medium was replaced by medium containing 100 μg/ml rifampicin and 40 μg/ml Cytosine β-D-arabinofuranoside (both from Sigma-Aldrich, München) to limit rMVA-T7 replication. After an additional 24 h, the culture medium was collected, clarified from cellular debris by centrifugation (4,700 rpm, 10 min, 4°C) and twice filtered through a membrane filter with a pore size of 0.2 μm to eliminate rMVA-T7 from the preparation. Next, fresh BHK-21 cells grown in T-75 flasks were inoculated with a 1:100 dilution of the passage 0 (P0) to amplify VSV* for virus stock production (P1).

Quantification of titers from VSV* stocks and cell culture supernatant was carried out on confluently grown BHK-21 cells seeded in 96-well plates. After removal of the cell culture supernatant, cells were inoculated with 10-fold serial dilutions of virus (diluted in serum-free medium). At 1 h post infection, the inoculum was removed and cells were overlaid with culture medium containing 2% methylcellulose (Sigma-Aldrich, München). After an incubation of 16–18 h, eGFP-positive cells were counted under the fluorescence microscope to determine viral titers (displayed as focus forming units per ml, ffu/ml). In addition, VSV* titers were also determined for HeLa cells as this cell line is less susceptible to VSV infection (~100x) and therefore titers determined on BHK-21 cells are not useful to calculate the optimal infectious dose for HeLa cells. To verify the authenticity of wt and mutant VSV-G in VSV*, viral RNA was isolated from P1 stocks and reverse-transcribed into cDNA using the SuperScript III First-Strand Synthesis System (ThermoFisher Scientific, Dreiech) according to the manufacturer’s protocol (for random hexamers). Next, a ~1,800 bp fragment was amplified with primers binding in the intergenic region upstream of VSV-G (forward) and the 5’ end of the eGFP ORF (reverse) using Phusion polymerase (ThermoFisher Scientific, Dreiech), separated by agarose gel electrophoresis and extracted from the gel by commercial kits (Macherey & Nagel, Düren), before being subjected to automated sequence analysis (SeqLab, Göttingen).

### Infection of cell lines with VSV

To assess the effect of directed tetherin expression on the spread of VSV*, 293T cells were transfected with increasing amounts of expression plasmid encoding for human tetherin. To avoid unspecific effects due to differences in the total DNA amounts of the tetherin vector, empty expression plasmid was used for equilibration. Cells transfected only with empty expression vector served as controls. At 16 h post transfection, the transfection medium was replaced by culture medium and the cells were further incubated for 24 h before they were infected. Additionally, Vero cells stably expressing human tetherin (Vero-Tetherin) or stably containing empty vector were used. In order to analyze the impact of siRNA-mediated knock-down of endogenous tetherin expression on VSV spread, HeLa cells were transfected with siRNA specific to human tetherin or scrambled siRNA (control) (both Santa Cruz, Dallas; 10 pM final concentration) using Lipofectamine2000 and OptiMEM medium (both from ThermoFisher Scientific, Dreieich). At 6 h post transfection, the transfection medium was replaced by culture medium and the cells were further incubated for 24 h before they were infected. For infection, VSV* stocks were diluted with serum-free medium to obtain the desired MOI and inoculated onto target cells for 1 h. Afterwards, the inoculum was removed and cells were washed with PBS before receiving fresh culture medium. Next, cells were further incubated and supernatants were collected for quantification of VSV* titers at different time points.

### Generation of VSV pseudotypes and transduction experiments

To analyze VSV-G-driven host cell entry, VSV vectors were pseudotyped with either VSV-G wt or mutants A133R and LXXXL. For this, 293T cells were transfected with the respective expression plasmids or empty plasmid as negative control. At 24 h post transfection, cells were inoculated with VSV-G trans-complemented VSV*ΔG that lacks the genetic information for VSV-G but codes for eGFP and firefly luciferase from two independent transcription units [42] (kindly provided by G. Zimmer) at an MOI of 3. At 1 h post infection, the inoculum was removed and the cells were washed with PBS. Next, medium containing a neutralizing antibody directed against VSV-G (I1, produced from CRL-2700 hybridoma cells, ATCC) was added to the cells and left for 1 h in order to neutralize residual input virus that had not entered the cells so far. Subsequently, the cells were again washed and further incubated with fresh culture medium for 16–18 h. Then, the supernatant was collected, clarified from cellular debris by centrifugation (4,700 rpm, 10 min, 4°C) and used for transduction experiments. For this, 293T cells were inoculated with identical volumes of the respective VSV pseudotypes and firefly luciferase activity in cell lysates was quantified at 20 h post inoculation using a plate luminometer (Hidex) and commercial substrates (PJK and Promega) as described elsewhere [43].

### Analysis of tetherin expression at the cell surface

For analysis of the surface expression of tetherin, 293T cells were cotransfected with tetherin plasmid and plasmid encoding viral antagonist or empty plasmid as negative control. At 48 h post transfection, the cells were washed and harvested in PBS. Expression of tetherin at the cell surface was detected by employing a tetherin-specific mouse monoclonal antibody (BioLegend) at a dilution of 1:50 and an Alexa 647-conjugated anti-mouse secondary antibody at a dilution of 1:100 (ThermoFisher Scientific, Dreieich). Subsequently, cells were fixed with 2% paraformaldehyde and staining was analyzed employing a LSR II Flow Cytometer (BD Biosciences, Heidelberg) and the FACS Diva software (BD Biosiences, Heidelberg). The data were further analyzed using the FCS Express 4 Flow research software (De Novo software).

### Quantification of cell viability

To ensure that directed tetherin expression did not lead to unwanted cytotoxic side-effects at the concentrations used, we analyzed cell viability employing the CellTitre-Glo kit (Promega, Mannheim). For this, 293T cells grown in 12-well plates were transfected with increasing amounts of tetherin expression plasmid or empty vector, which was also used to equilibrate total DNA amounts. At 16 h post transfection, the transfection medium was replaced by fresh culture medium and the cells were further incubated for 24 h. Next, the cell culture supernatants were removed and 200 μl of the CellTitre-Glo reagent were added. After an incubation period of 10 min at room temperature, 100 μl of the lysates were transferred into a white, opaque-walled 96-well plate, before luminescence was recorded using a plate luminometer (Plate Chameleon V, Hidex, Turku). All samples were analyzed in triplicates. For normalization, viability of cells transfected only with empty expression vector was set as 100% and relative viability of tetherin-transfected cells was calculated.

### Statistical data analysis

If not explicitly stated in the figure legends, unpaired and paired Student t-tests were performed to analyze data pairs originating from individual experiments or mean data of multiple experiments, respectively. One-way ANOVA with Bonferroni post-test analysis was carried out for combined comparison of multiple groups (*, p ≤ 0.05; **, p ≤ 0.005; ***, p ≤ 0.001).

## Results

### VSV-G antagonizes human and porcine tetherin

**Fig 1:**
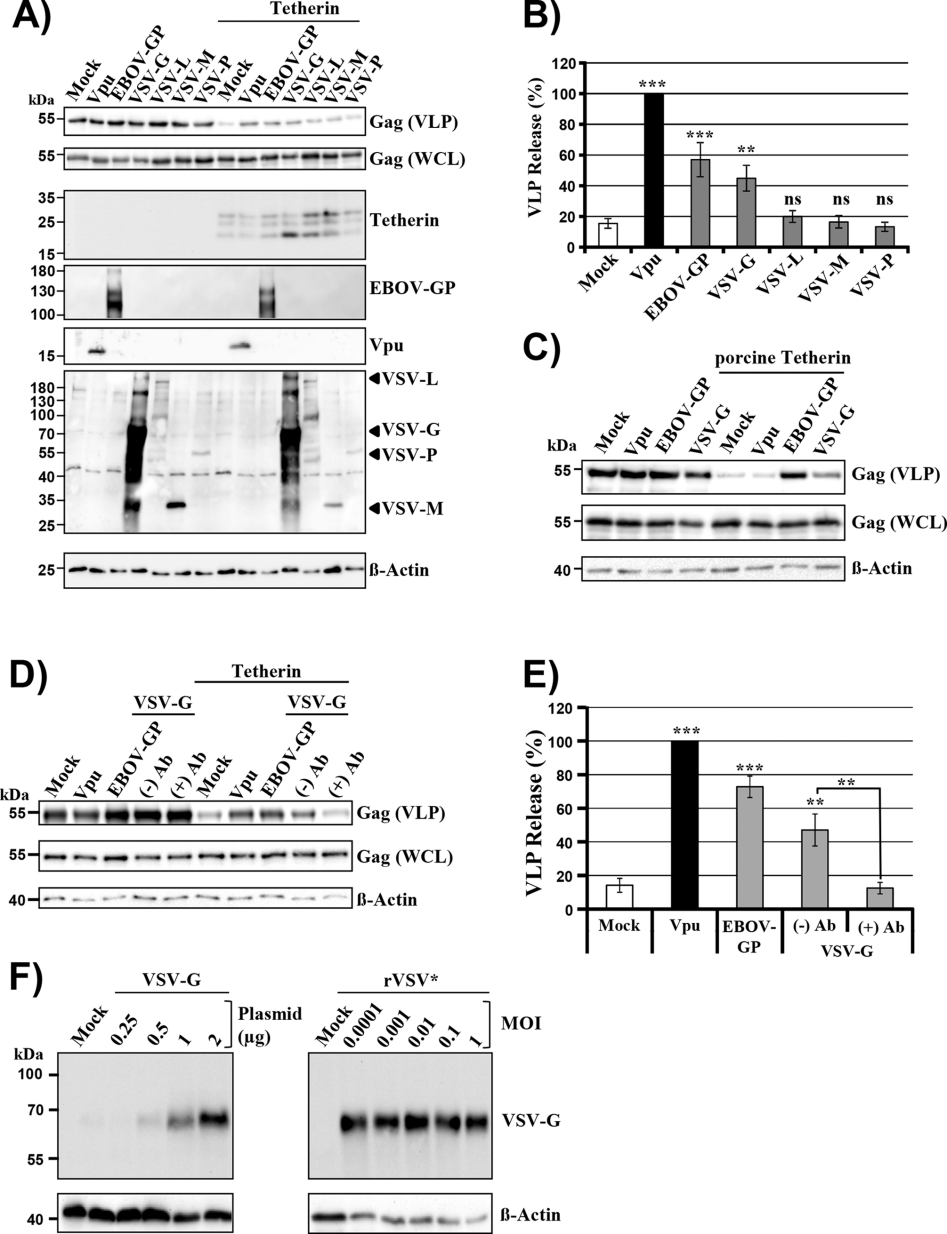
VSV-G antagonizes human and porcine tetherin. (A) 293T cells were transiently transfected with plasmids encoding HIV-Gag, tetherin and the indicated viral proteins or empty plasmid as negative control (Mock). HIV-1 Vpu and EBOV-GP were employed as positive controls for tetherin antagonism. The presence of Gag in supernatants and cell lysates was determined by Western blot analysis using an anti-Gag antibody. Expression of tetherin was detected using anti-c-Myc antibody while rabbit sera were employed to detect expression of EBOV-GP, Vpu and VSV proteins. Detection of ß-actin in cell lysates served as loading control. Similar results were obtained in four separate experiments. (B) The average of eight independent experiments conducted as described for panel (A) and quantified via the ImageJ program is shown. Release of Gag from cells coexpressing Vpu and tetherin was set to 100%. One-way ANOVA with Bonferroni post-test analysis was performed to test whether signals measured in the presence of tetherin antagonists were statistically different from those detected in the absence of antagonists (Mock). (C) The same experiment was performed as in (A) but instead of human tetherin porcine tetherin was examined. The results of a representative experiment are shown and were confirmed in three separate experiments. (D) 293T cells were transfected as described for panel (A) and at 16 h post transfection the medium was replaced by fresh culture medium supplemented with ((+)Ab) or without ((-)Ab) hybridoma supernatant containing VSV neutralizing antibody at a final concentration of 1:1,000. (E) The average of four independent experiments conducted as described for panel (D) and quantified via the ImageJ program is presented. Release of Gag from cells coexpressing Vpu and tetherin was set to 100%. One-way ANOVA with Bonferroni post-test analysis was performed to test whether differences between signals obtained from cells expressing viral tetherin antagonists and cells expressing no antagonist (Mock), or between cells expressing VSV-G and treated with or without anti-VSV-G antibody (**, p ≤ 0.005; ***, p ≤ 0.001) were statistically significant. (F) 293T cells were transiently transfected with the indicated amounts of plasmid encoding VSV-G. At 48 h post transfection the expression of VSV-G was determined via Western blot using anti-VSV-G hybridoma supernatant (left panel). In parallel, 293T cells were infected with VSV using the indicated multiplicities of infection (MOI) and VSV-G expression was examined by Western blot at 24 h post infection using anti-VSV-G antibody (concentrated supernatants from hybridoma CL-2700). The results of a representative experiment are shown and were confirmed in three separate experiments.

In order to analyze tetherin antagonism by VSV proteins, we employed a previously reported virus-like particle (VLP) release assay, which measures release of HIV-Gag-based VLPs from transfected 293T cells and its blockade by tetherin [27,32]. Release of VLPs was markedly diminished upon coexpression of tetherin and tetherin’s antiviral activity was blocked by the well-established tetherin antagonists Vpu and EBOV-GP (Fig 1A and 1B), as expected. In addition, VSV-G but not VSV-L, M (mutant M(ncp), [33]) and P interfered with tetherin’s antiviral activity, although less efficiently than Vpu and EBOV-GP (Fig 1A and 1B). Finally, none of the viral proteins tested modulated VLP release from tetherin-negative control cells, indicating that the effects observed in tetherin-positive cells were specific. Thus, VSV-G can antagonize tetherin in transfected cells, although with reduced efficiency as compared to EBOV-GP and Vpu.

The studies described above were conducted with human tetherin. Since VSV can infect livestock, we next analyzed if VSV-G also antagonizes pig-derived tetherin. We found that Vpu failed to antagonize porcine tetherin (Fig 1C), in keeping with the well-established notion that the anti-tetherin activity of Vpu is highly species specific [44–46]. In contrast, both EBOV-GP and VSV-G rescued VLP release from blockade by porcine tetherin (Fig 1C), indicating that VSV-G might be able to promote viral spread in infected pigs by antagonizing tetherin.

### Evidence that tetherin antagonism by VSV-G can be blocked by a G-protein specific antibody and is not due to VSV-G overexpression

We next sought to investigate whether tetherin counteraction by VSV-G can be inhibited by antibodies. This question was triggered by our previous observation that antibodies directed against EBOV-GP can interfere with GP-mediated tetherin counteraction [32]. To this end, we added supernatant of hybridoma CRL-2700 (which secretes the VSV neutralizing antibody I1) to cells expressing VSV-G and releasing VLPs in the presence and absence of tetherin. The hybridoma supernatant did not modulate VLP release from tetherin-negative control cells coexpressing VSV-G but abrogated VLP-release from cells coexpressing tetherin and VSV-G (Fig 1D and 1E). We cannot exclude that the antibody triggered VSV-G internalization and a control antibody remains to be tested. However, the results available at present suggest that antibodies directed against VSV-G might not only block viral entry into target cells but may also interfere with tetherin antagonism. Moreover, our findings indicate that the ectodomain of VSV-G plays an important role in tetherin counteraction.

We next investigated whether the VSV-G expression levels attained in transfected cells exceeded those found in infected cells, which would suggest that tetherin counteraction by VSV-G could have been due to overexpression. For this, VSV-G expression levels in cells transfected to express VSV-G and cells infected with a recombinant VSV encoding GFP (VSV*) were compared. Cells were harvested at the same time points at which VLP release and (Figs 1A–1E and 5E and 5F) and VSV release (Fig 3A–3C and Fig 6A–6D) from tetherin-positive cells were examined within functional assays. The immunoblot revealed that less G-protein was expressed in transfected as compared to infected cells (Fig 1F), indicating that tetherin antagonism was not due to overexpression. In this context, it should be noted that the lack of correlation between infectious dose and G-protein expression levels resulted from the fast replication kinetics of VSV, which led to 100% infected cells at the time of analysis. Moreover, the reduced signals for actin in VSV- versus mock-infected cells can be attributed to the well-established cytotoxic effects associated with VSV infection [33,47,48]. Collectively, the results discussed above show that VSV-G can antagonize tetherin at expression levels below that attained in infected cells, although counteraction is less efficient as compared to Vpu and EBOV-GP.

### VSV-G does not interfere with tetherin expression

**Fig 2:**
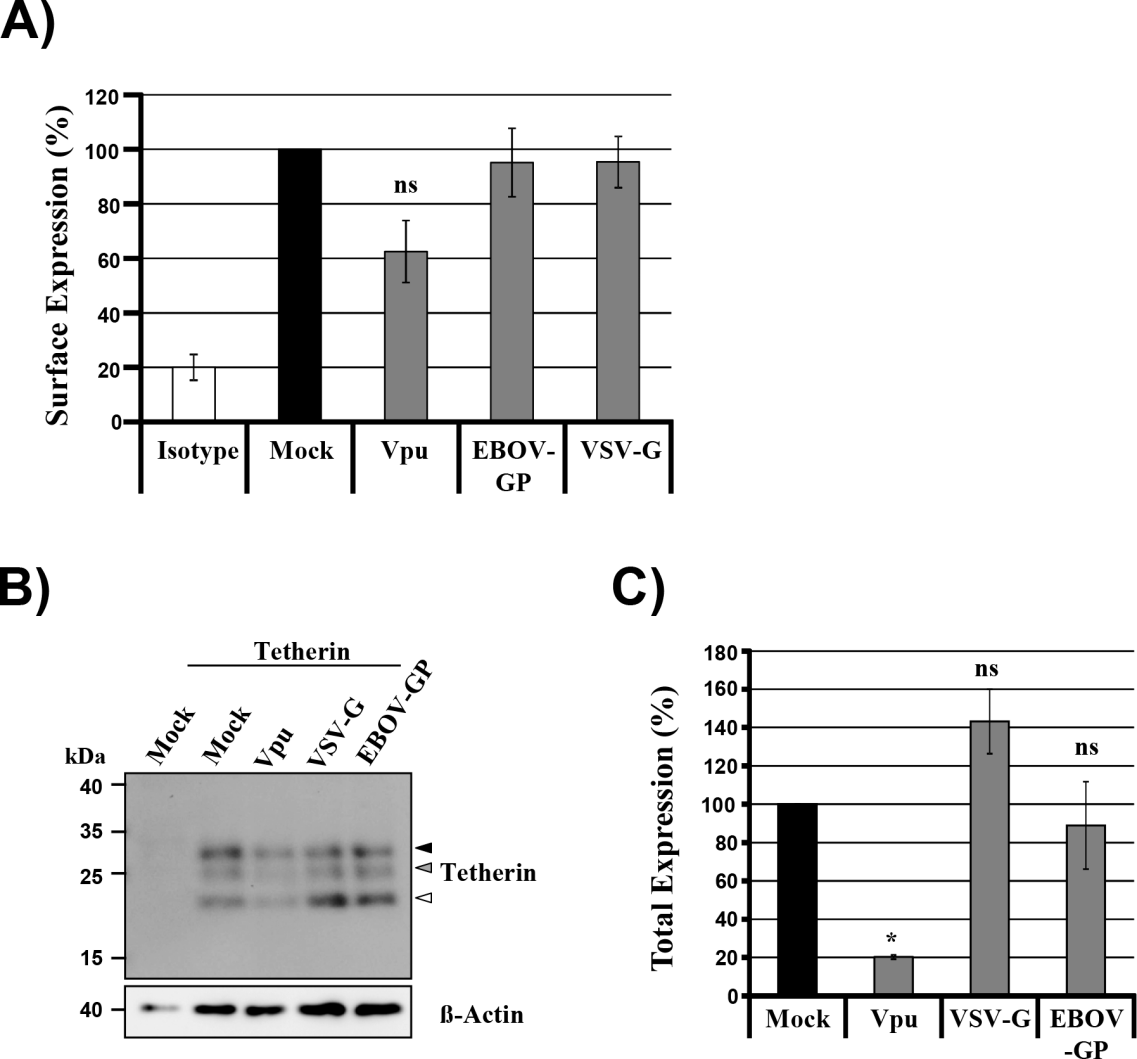
VSV-G does not alter expression levels or cell surface localization of tetherin. (A) Plasmids encoding tetherin and the indicated viral tetherin antagonists were transiently transfected into 293T cells. Transfection of empty plasmid (Mock) served as negative control. At 48 h post transfection, cells were harvested and stained for surface expression of tetherin using anti-tetherin antibody and Alexa-647 coupled secondary antibody. Staining was analyzed by flow cytometry. The graph shows relative surface expression values from three independent experiments, for which expression of tetherin on the surface of cells not expressing any antagonist (Mock) was set as 100%. Error bars indicate standard error of the mean (SEM). (B) 293T cells were transfected with plasmids encoding tetherin with an N-terminal c-Myc-tag and the indicated viral tetherin antagonists. At 48 h post transfection, tetherin expression in cell lysates was detected by Western blot analysis, using anti-c-Myc antibody. Detection of β-actin served as loading control. The results were confirmed in three separate experiments. Arrow heads indicate bands exhibiting the molecular weight expected for unglycosylated (white), partially (grey) and fully (black) N-glycosylated tetherin. (C) Quantification of four experiments performed as described for panel (B). The intensities measured for all tetherin signals with a molecular weight between 17 and 30 kDa were added to yield total tetherin expression. Tetherin expression in the absence of antagonist (mock) was set to 100%. Error bars indicate SEM. One-way ANOVA with Bonferroni post-test analyses were performed for panels A and C to test for statistically significant differences between samples with and without (Mock) antagonist (*, p ≤ 0.05).

Tetherin antagonism by viral proteins is usually due to removal of tetherin from the site of viral budding, the plasma membrane [20,22]. In order to investigate whether VSV-G also interferes with tetherin expression at the plasma membrane, we performed flow cytometry with cells transfected to coexpress tetherin and viral antagonists. Coexpression of Vpu but not EBOV-GP reduced tetherin surface levels (Fig 2A), as expected from previous reports [27,28]. In contrast, coexpression of VSV-G did not modulate surface levels of tetherin (Fig 2A). In agreement with these findings, neither VSV-G nor EBOV-GP coexpression reduced tetherin levels in cell lysates while a marked decrease in tetherin levels was observed upon coexpression of Vpu (Fig 2B and 2C). Thus, VSV-G, like EBOV-GP, employs a mechanism different from that of Vpu for tetherin counteraction. Upon coexpression of VSV-G and EBOV-GP, a band with the size expected for unglycosylated tetherin accumulated (Fig 2B), which is known to exert reduced antiviral activity as compared to the fully glycosylated form [21]. Therefore, it is conceivable that VSV-G and EBOV-GP inhibit tetherin by interfering with its N-glycosylation, similarly as reported for SARS-coronavirus ORF7a protein [49].

### Tetherin blocks release of infectious VSV

**Fig 3:**
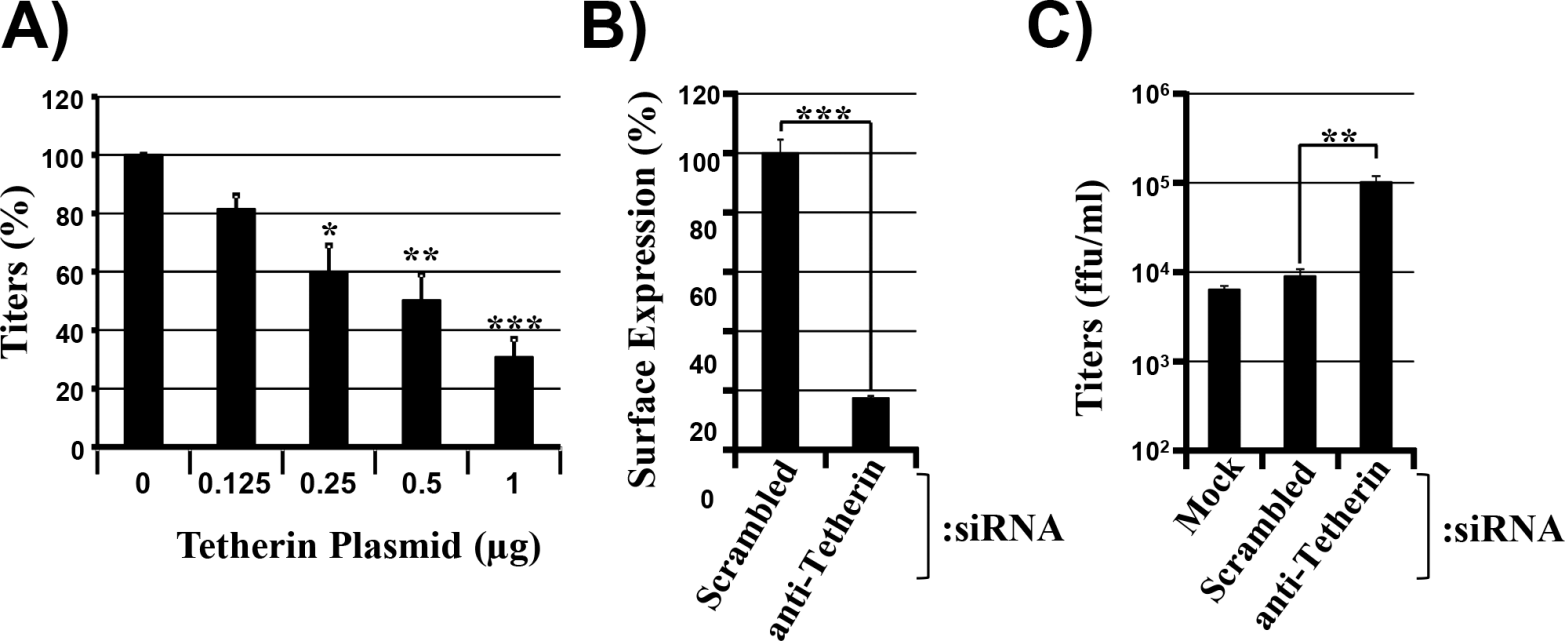
Tetherin inhibits VSV spread. (A) 293T cells were transiently transfected with rising amounts of plasmid encoding human tetherin. At 24 h post transfection cells were infected with VSV at an MOI of 0.001 washed and viral titers present in culture supernatants were determined at 8 h post infection. Titers measured in the absence of tetherin were set to 100%. The average of six independent experiments is shown, error bars indicate standard error of the mean (SEM). Unpaired two-tailed Student’s t-test was used to examine whether differences in titers obtained from cells transiently expressing tetherin versus cells transfected with empty expression vector are of statistical significance (*, p ≤ 0.05; **, p ≤ 0.005; ***, p ≤ 0.001). (B) HeLa cells were transfected with the indicated siRNAs. At 48 h post transfection, cells were harvested and stained with anti-tetherin antibody. Cell staining was then analyzed by flow cytometry. The results of a single representative experiment carried out with triplicate samples are shown, in which tetherin surface expression levels in cells transfected with control siRNA (scrambled) were set as 100%. Error bars indicate standard deviation (SD). An unpaired two-tailed Student’s t-test was used to assess statistical significance (***, p ≤ 0.001). (C) HeLa cells were transfected with the indicated siRNAs and at 24 h post transfection cells were infected with VSV at an MOI of 0.005 At 12 h post infection, viral titers in culture supernatants were determined. The results of a single representative experiment conducted with triplicate samples are shown and were confirmed in two separate experiments. Error bars indicate standard deviation. To test whether differences in VSV titers measured for cells transfected with control (scrambled) and tetherin-specific siRNA were statistically significant, an unpaired two-tailed Student’s t-test was performed (**, p ≤ 0.05).

Weidner and colleagues reported that VSV spread can be efficiently blocked by directed expression of tetherin [18], and a subsequent study demonstrated that tetherin mainly inhibits cell-free but not cell-associated VSV spread [17]. We sought to confirm these findings and to establish a cellular system which allows investigating the potential contribution of VSV-G-mediated tetherin counteraction to viral spread. For this, we transfected 293T cells with rising amounts of tetherin plasmid, infected the cells with VSV and quantified the number of infectious units present in culture supernatants at 8 h post infection. We observed a modest but dose-dependent reduction of viral titers upon transfection of increasing amounts of tetherin-plasmid (Fig 3A) in the absence of appreciable cytotoxic effects (S1 Fig). Moreover, siRNA-mediated knock-down of endogenous tetherin expression in HeLa cells reduced the amount of cell surface associated tetherin (Fig 3B) and increased viral titers roughly 10-fold as compared to cells transfected with scrambled siRNA or mock transfected cells (Fig 3C). Collectively, these findings confirm that VSV is inhibited by tetherin and indicate that knock-down of tetherin expression in HeLa cells affords the opportunity to examine whether VSV-G-mediated tetherin antagonism is operative in the context of infected cells.

### A GXXXG motif in the transmembrane domain of VSV-G contributes to tetherin antagonism in transfected cells but is dispensable for VSV spread in tetherin-positive cells

**Fig 4:**
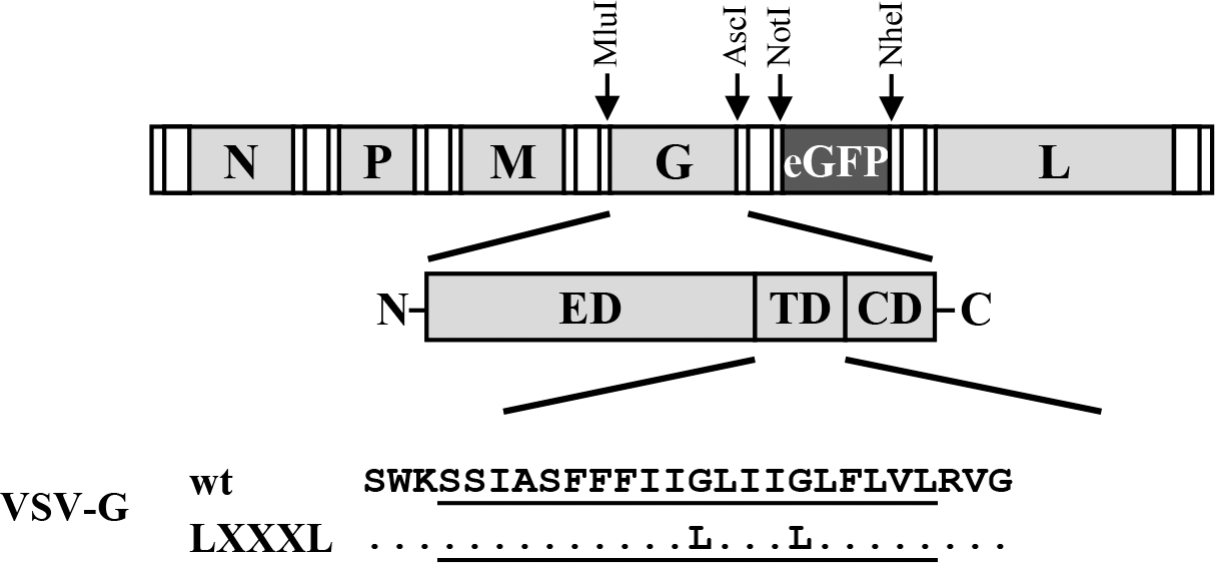
Mutagenesis of the GXXXG motif in the transmembrane domain of VSV-G. Schematic representation of the VSV* genome in which the authentic viral open reading frames (ORFs, VSV-N, -P, -M, -G and -L; light grey) are surrounded by non-translated regulatory elements (white). The additional ORF for eGFP (dark grey) was inserted between the ORFs for VSV-G and -L (restriction sites used for cloning and further modification of the genome are highlighted). The transmembrane domain (underlined amino acids) of VSV-G wt contains a GXXXG motif (amino acid residues 473–477) that has been mutated to LXXXL.

**Fig 5:**
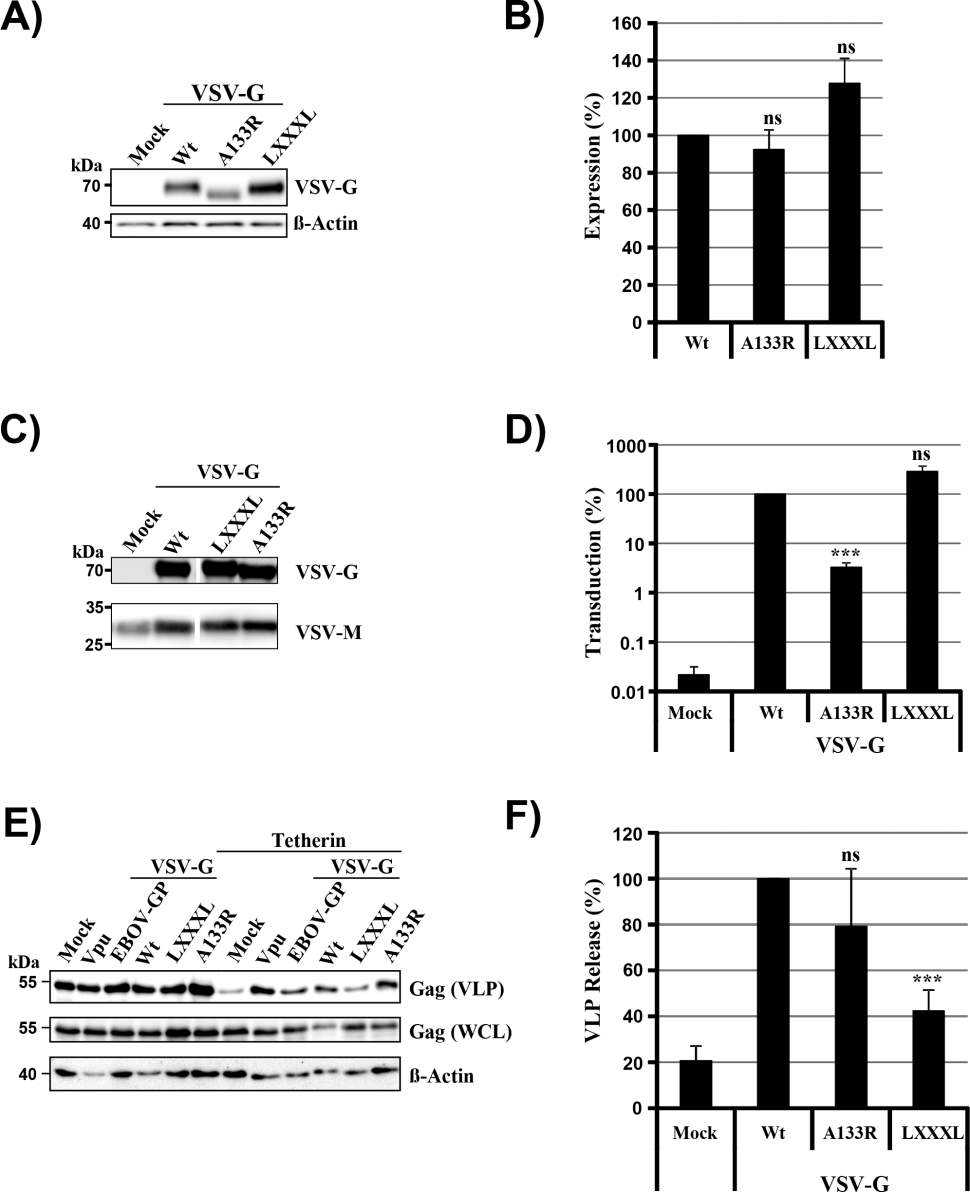
The GXXXG motif in the transmembrane domain of VSV-G is required for tetherin antagonism in transfected cells. (A) 293T cells were transfected with plasmids encoding the indicated glycoproteins or mock-transfected. Expression was determined by Western blot analysis using anti-VSV-G antibody (concentrated supernatants from hybridoma CL-2700). Detection of β-actin expression served as loading control. (B) The average of four independent experiments conducted as described for panel (A) and quantified via the ImageJ program is presented. Expression of VSV-G wt was set to 100%. (C) Incorporation of VSV-G wt and mutant LXXXL into VSV pseudotypes was investigated by Western blot analysis using an anti-VSV-G antibody (concentrated supernatants from hybridoma CL-2700). To ensure that similar amounts of pseudotypes were analyzed, levels of particle-associated M proteins were determined using an anti-VSV-M antibody. Pseudotypes harboring no glycoprotein (Mock) served as negative control. The results of a single immunoblot are shown from which irrelevant lanes were cut out. Similar results were obtained in two separate experiments. (D) 293T cells were transduced with equal volumes of the VSV pseudotypes described in panel (C). At 24 h post transduction, luciferase activity in cell lysates was measured. Transduction driven by VSV-G wt was set as 100%. The average of three independent experiments is shown. Error bars indicate SEM. (E) 293T cells were cotransfected with plasmids encoding Gag, the indicated glycoproteins and tetherin. Expression of Gag in supernatants and cell lysates was determined by Western blot. Detection of β-actin expression served as loading control. (F) The average of three independent experiments conducted as described for panel (E) and quantified via the ImageJ program is presented. Error bars indicate standard error of the mean (SEM). Release of Gag from cells coexpressing the highest amount of VSV-G and tetherin was set to 100%. For all graphs (B, D, F), paired two-tailed Students’ t-tests were performed to assess whether differences between VSV-G wt and LXXXL mutant were of statistical significance.

**Fig 6:**
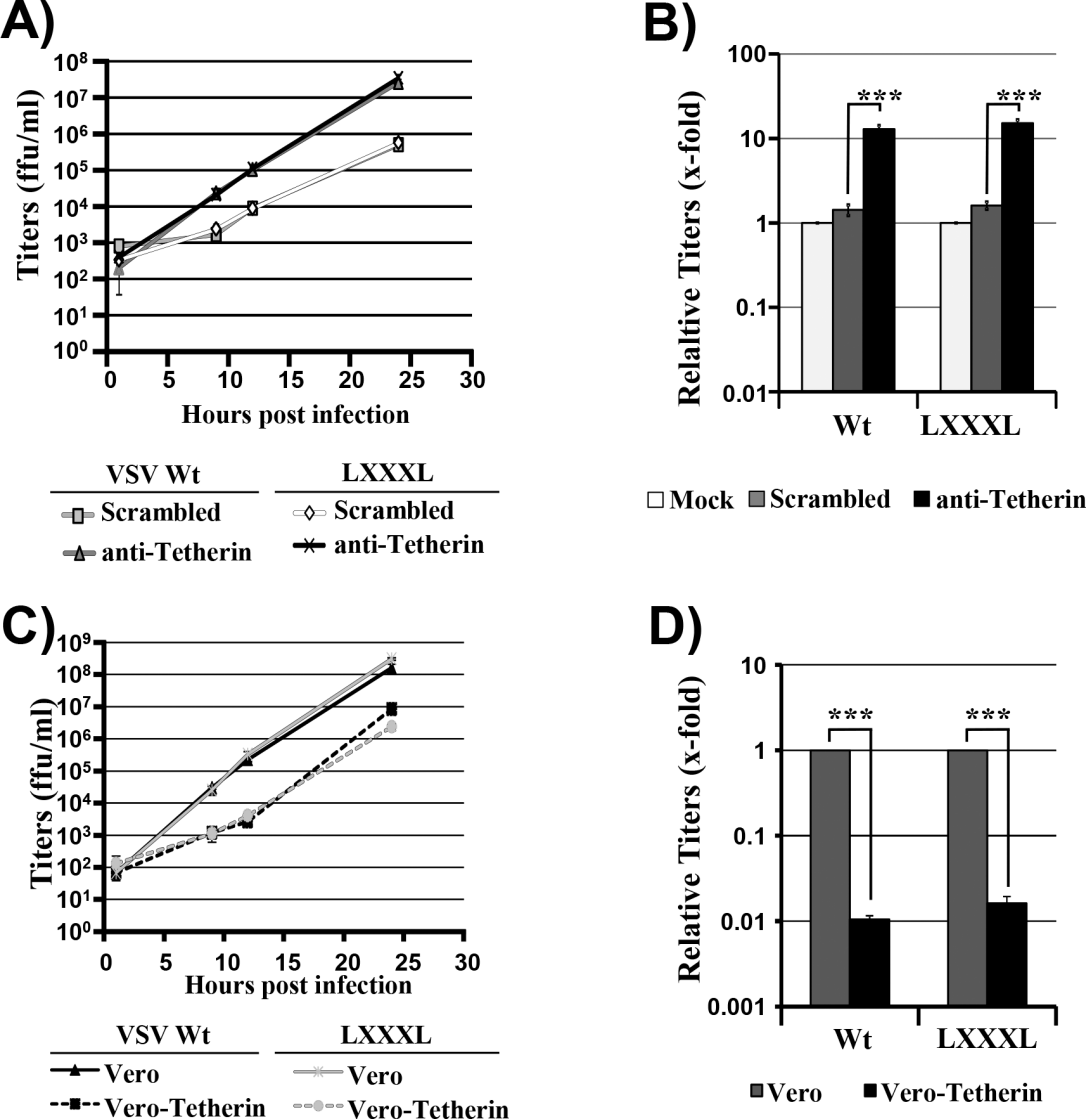
The GXXXG motif is dispensable for viral spread in tetherin-positive cells. (A) HeLa cells were transfected with the indicated siRNAs and subsequently infected with VSV wt or LXXXL mutant at an MOI of 0.005 for 1 h. Virus titers in culture supernatants were determined at the indicated time points post infection. The results of a single representative experiment carried out with triplicate samples are shown and were confirmed in two separate experiments. (B) The experiment was carried out as described for panel (A) but relative titers measured at 12 h post infection are shown. The results represent the average of three independent experiments performed with triplicate samples. Titers measured for untransfected control cells were set to 1. Error bars indicate standard error of the mean (SEM). (C) Vero cells stably expressing human tetherin (Vero-Tetherin) or stably containing empty vector (Vero) were infected with VSV wt or mutant LXXXL. Viral titers in culture supernatants were determined at the indicated time points post infection. The results of a single representative experiment carried out with triplicate samples are shown and were confirmed in two separate experiments. (D) The experiment was carried out as described for panel (C) but relative titers measured at 12 h post infection are shown. The results represent the average of three independent experiments performed with triplicate samples. Titers obtained from the respective control Vero cells were set to 1. Error bars indicate SEM. One-way ANOVA with Bonferroni post-test analyses were performed (B and D) to test statistical significance between selected groups (***, p ≤ 0.001).

Having established that VSV-G can antagonize tetherin upon directed expression and that VSV spread is reduced but not abrogated by tetherin, we finally sought to determine whether VSV-G-mediated tetherin antagonism contributes to viral spread. For this endeavor, we needed to identify a mutation in VSV-G that selectively interferes with tetherin antagonism. To this end, we tested a VSV-G mutant in which a GXXXG motif in the transmembrane domain was changed to LXXXL (Fig 4). This mutation was chosen, since our unpublished results indicate that a GXXXA motif in the transmembrane domain of EBOV-GP contributes to tetherin antagonism. In addition, we analyzed VSV-G mutant A133R. This mutation is known to interfere with viral entry by rendering VSV-G defective for membrane fusion [50]. Both mutation A133R and mutation of the GXXXG motif (mutant LXXXL) were compatible with robust G-protein expression (Fig 5A and 5B) and particle incorporation (Fig 5C). Moreover, mutation of the GXXXG motif (mutant LXXXL) did not interfere with host cell entry of VSV-G pseudotypes (Fig 5D). In contrast, mutation A133R markedly reduced entry efficiency (Fig 5D), as expected [50]. Finally, mutant A133R was able to counteract tetherin while mutant LXXXL failed to efficiently antagonize tetherin (Fig 5E and 5F). Thus, mutation of the GXXXG motif afforded an opportunity to investigate whether G-protein-mediated tetherin antagonism contributes to viral spread. For this, the LXXXL mutation was introduced into the VSV genome, employing a reverse genetics system, and infectious VSV was rescued. Infection of siRNA-transfected HeLa cells with VSV wt and mutant LXXXL at equal MOI revealed that knock-down of tetherin expression increased VSV wt release, as expected. However, a comparable rescue of viral release was observed for the LXXXL mutant (Fig 6A and 6B). Moreover, spread of VSV wt and mutant LXXXL was comparably reduced by directed expression of tetherin in Vero cells (Fig 6C and 6D), suggesting that tetherin antagonism by VSV-G might not appreciably contribute to viral spread in tetherin-positive cells.

## Discussion

Tetherin and other effector proteins of the IFN system are responsible for the establishment of an antiviral state in IFN exposed cells [12,13]. Many viruses evolved countermeasures against these antiviral effectors by either multi-functionalizing their structural proteins or by acquiring non-structural proteins with antagonistic activity. Whether VSV, which is sensitive to blockade by tetherin [17,18], encodes an antagonist that allows residual viral spread in tetherin-positive cells is unknown. Here, we show that the surface protein of VSV, VSV-G, can antagonize tetherin, at least upon directed expression. Tetherin counteraction by VSV-G did not involve modulation of tetherin expression and was partially dependent on a GXXXG motif in the VSV-G transmembrane domain. However, mutation of the GXXXG motif in the context of infectious VSV did not affect viral spread in tetherin-positive cells. Thus, VSV-G is a novel tetherin antagonist but the potential contribution of its antagonistic activity to viral spread in tetherin-positive cells requires further investigation.

Previous studies reported that directed expression of tetherin in NIH 3T3 cells and 293 cells markedly diminished viral release [17,18]. However, tetherin expression failed to reduce viral release to background levels in both studies, although tetherin was expressed at high levels, which leaves the possibility that VSV encodes a modestly active tetherin antagonist. In keeping with such a scenario, we found that VSV-G antagonizes human and pig tetherin but does so less efficiently than Vpu and EBOV-GP. Tetherin antagonism could only be demonstrated in the context of directed VSV-G expression in the present study. Nevertheless, tetherin counteraction was not due to G-protein overexpression, indicating that VSV-G could block tetherin in infected cells, as discussed below.

Several viral tetherin antagonists inhibit tetherin by interfering with its expression or appropriate cellular localization. For instance, Vpu can interfere with the anterograde transport of tetherin [51,52] and directs tetherin towards lysosomal degradation [23–25], which ultimately results in reduced tetherin levels at the cell surface. In contrast, VSV-G did not modulate total tetherin expression or tetherin levels at the cell surface. These findings are similar to those previously reported for EBOV-GP [27,28], which antagonizes tetherin by a so far unknown mechanism. Moreover, VSV-G, like EBOV-GP, was active against diverse tetherins, as demonstrated by the counteraction of porcine tetherin, which shares only 48% sequence identity with human tetherin. Finally, our finding that antibody I1 directed against the VSV-G ectodomain interferes with tetherin antagonism recapitulates findings previously made for EBOV-GP [32] and suggests that both VSV-G and EBOV-GP depend on their ectodomains for tetherin counteraction. How VSV-G (and EBOV-GP) counteracts tetherin remains unknown and at present a role of direct interactions (potentially disrupted by anti-ectodomain antibodies) cannot be discounted. Our finding that coexpression of VSV-G resulted in increased generation of unglycosylated tetherin, which is known to display little antiviral activity [21], suggests that VSV-G might block tetherin, at least in part, by interfering with its posttranslational modification. A similar inhibitory strategy has previously been reported for the SARS-coronavirus ORF7A protein [49] but the underlying mechanism is incompletely understood.

In order to get initial insights into the contribution of VSV-G-mediated tetherin antagonism to VSV release from tetherin-positive cells, we examined previously characterized mutations in the ectodomain (A133R) and the transmembrane domain (G473L and G477L, mutant LXXXL) of VSV-G for their effect on tetherin antagonism. A133R markedly reduced VSV-G-driven entry, as expected [50], but did not affect tetherin antagonism. In contrast, mutation of the GXXXG motif in the transmembrane domain had no impact on entry but reduced tetherin antagonism by roughly 60%, as discussed below. The finding that the GXXXG motif was dispensable for VSV-G-driven virus-cell fusion was unexpected, since the same motif has been reported to be required for cell-cell fusion induced by exposure of VSV-G expressing cells to low pH [53]. In this context, it is important to state that the integrity of the LXXXL mutations after amplification of the rescued viruses in BHK-21 cells (passage 1 stocks) has been confirmed by sequencing. Therefore, the GXXXG motif seems to be important for VSV-G-driven cell-cell but not virus-cell fusion and the underlying reasons remain to be determined.

The finding that the GXXXG motif is required for tetherin antagonism is in keeping with our unpublished observation that a GXXXA motif in the EBOV-GP transmembrane domain, which has been reported to be important for EBOV-GP-mediated cellular detachment [54], is required for full tetherin antagonism by EBOV-GP. Although mutation of the GXXXG motif in VSV-G reduced tetherin antagonism in transfected cells, it had no impact on viral spread in HeLa cells, which express endogenous tetherin [19], and Vero cells, which were engineered to express tetherin. These observations could indicate that VSV-G-mediated tetherin antagonism is not operative in VSV infected cells. In fact, one could speculate that in infected cells the interaction between VSV-G and other viral proteins, which is required for assembly of progeny particles [55], compromises the ability of VSV-G to counteract tetherin. Alternatively, it is conceivable that the modest reduction of tetherin antagonism observed in transfected cells upon mutation of the GXXXG motif precluded detection of anti-tetherin effects in infected cells. The identification of a mutation in VSV-G that selectively and efficiently reduces tetherin antagonism is required to address this question.

Collectively, our results and published data indicate that VSV-G, EBOV-GP [26] and possibly other viral glycoproteins can interfere with tetherin’s antiviral activity via a mechanism that is relatively independent of the tetherin sequence. Glycoprotein-mediated interference with tetherin’s N-glycosylation status might contribute to this effect. Moreover, our results indicate that findings made for tetherin-antagonism in transfected cells might not always adequately reflect the situation in infected cells. Finally, our findings suggest the possibility that VSV might acquire robust tetherin antagonism upon infection of humans.

## Supporting information

S1 Fig Directed tetherin expression is not associated with major cytotoxic effects. 293T cells were transfected with increasing amounts of expression vector for human tetherin. Empty vector was used for equilibration of total DNA amounts. At 48 h post transfection, intracellular ATP levels were quantified. The average of three independent experiments performed with triplicate samples is shown, error bars indicate standard error of the mean (SEM). A paired two-tailed Student’s t-test was used to examine whether differences in cell viability between cells transfected with empty vector (0 μg tetherin plasmid) and tetherin-expressing cells were of statistical significance (ns, not significant; *, p ≤ 0.05).
(PDF)
