# Supplementary material for: The glycoprotein of vesicular stomatitis virus promotes release of virus-like particles from tetherin-positive cells
Source: PLoS One. 2017 Dec 7;12(12):e0189073. doi: 10.1371/journal.pone.0189073 (PMC5720808; doi:10.1371/journal.pone.0189073)
Supplement: S1 Fig — 293T cells were transfected with increasing amounts of expression vector for human tetherin. Empty vector was used for equilibration of total DNA amounts. At 48 h post transfection, intracellular ATP levels were quantified. The average of three independent experiments performed with triplicate samples is shown, error bars indicate standard error of the mean (SEM). A paired two-tailed Student’s t-test was used to examine whether differences in cell viability between cells transfected with empty vector (0 μg tetherin plasmid) and tetherin-expressing cells were of statistical significance (ns, not significant; *, p ≤ 0.05). (PDF) [file pone.0189073.s001.pdf]

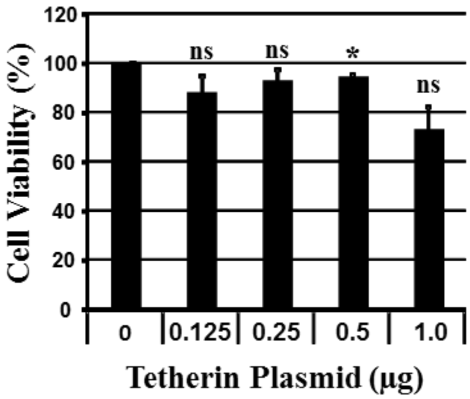

S1 Fig. Directed tetherin expression is not associated with major cytotoxic effects. 293T cells were transfected with increasing amounts of expression vector for human tetherin. Empty vector was used for equilibration of total DNA amounts. At 48 h post transfection, intracellular ATP levels were quantified. The average of three independent experiments performed with triplicate samples is shown, error bars indicate standard error of the mean (SEM). A paired two-tailed student's t-test was used to examine whether differences in cell viability between cells transfected with empty vector (0 µg tetherin plasmid) and tetherin-expressing cells were of statistical significance (ns, not significant; *, $p \leq 0.05$).
